# Geniculo-Cortical Projection Diversity Revealed within the Mouse Visual Thalamus

**DOI:** 10.1371/journal.pone.0144846

**Published:** 2016-01-04

**Authors:** Marcus N. Leiwe, Aenea C. Hendry, Andrew D. Bard, Stephen J. Eglen, Andrew S. Lowe, Ian D. Thompson

**Affiliations:** 1 MRC Centre for Developmental Neurobiology, King’s College London, Guy’s Campus, London, United Kingdom; 2 Cambridge Computational Biology Institute, Department of Applied Mathematics and Theoretical Physics, University of Cambridge, Cambridge, United Kingdom; NIH/NEI, UNITED STATES

## Abstract

The mouse dorsal lateral geniculate nucleus (dLGN) is an intermediary between retina and primary visual cortex (V1). Recent investigations are beginning to reveal regional complexity in mouse dLGN. Using local injections of retrograde tracers into V1 of adult and neonatal mice, we examined the developing organisation of geniculate projection columns: the population of dLGN-V1 projection neurons that converge in cortex. Serial sectioning of the dLGN enabled the distribution of labelled projection neurons to be reconstructed and collated within a common standardised space. This enabled us to determine: the organisation of cells within the dLGN-V1 projection columns; their internal organisation (topology); and their order relative to V1 (topography). Here, we report parameters of projection columns that are highly variable in young animals and refined in the adult, exhibiting profiles consistent with shell and core zones of the dLGN. Additionally, such profiles are disrupted in adult animals with reduced correlated spontaneous activity during development. Assessing the variability between groups with partial least squares regression suggests that 4–6 cryptic lamina may exist along the length of the projection column. Our findings further spotlight the diversity of the mouse dLGN–an increasingly important model system for understanding the pre-cortical organisation and processing of visual information. Furthermore, our approach of using standardised spaces and pooling information across many animals will enhance future functional studies of the dLGN.

## Introduction

Visual information passes from the retina to the primary visual cortex (V1) via the dorsal lateral geniculate nucleus (dLGN). The textbook view of the mouse dLGN is generally one of a simple relay nucleus, with no discrete laminar organisation, and connections from retinal ganglion cells (RGCs) to dLGN neurons exhibiting one-to-one relationships. However, growing evidence collectively challenges such consensus. For instance, functional direction-selective RGC types (DS-RGCs) with known molecular identities revealed a superficial cryptic lamination of the mouse dLGN [1,2] that is coincident with the calbindin positive shell [3]. Targeted functional investigations of the dLGN shell also revealed orientation-selective (OS) responses suggesting a local emergent property with unique projections to superficial laminae of V1 [4–7]. Collectively, these studies suggest the mouse dLGN has functionally specialised, parallel retino-geniculo-cortical pathways [8] and renews interest in the exact anatomical and functional organisation of the mouse dLGN. Understanding the form and organisation of the dLGN appears critical to understanding mouse visual processing.

The mouse dLGN is topographically ordered, with neighbouring RGC projections innervating adjacent regions within the dLGN. Such topographic organisation also maps to V1 such that a focal injection of retrograde tracer agent within V1 results in a labelled column of dLGN projection neurons that span the nucleus. Given the small size, convoluted three-dimensional structure, and inaccessibility of the mouse dLGN; the form and organisation of projection columns is poorly understood. To probe the organisation of RGC inputs and functional outputs of the mouse dLGN, it is critically important to know how projection columns traverse and are organised across the dLGN. The reconstruction of the mouse dLGN from serial histological sections following V1 tracer-injections and placing it within a standardised dLGN space, has enabled such a quantitative assessment of parameters that define projection columns within the neonatal (P6 and P12) and adult wild type dLGN (C57/BL6J) as well as a transgenic line with altered visual drive during development; β2^-/-^mice which lack the β2 sub-unit of the nicotinic acetylcholine receptor [9]. We report a surprising degree of anatomical diversity associated with columns of dLGN neurons projecting to V1. Analyses of diversity across development, suggests that a complex multi-laminar cryptic organisation may exist within the mouse dLGN. We provide an estimate as to the degree of potential laminar complexity within the dLGN.

## Materials and Methods

### Animals

Procedures were approved by the local Animal Care and Use Committee (King’s College London) and carried out in accordance with the Animals (Experimental Procedures) Act, 1986, under licence from the United Kingdom Home Office. Experiments were conducted on C57/BL6J mice of either sex (Harlan, UK) maintained on a 12/12 hour light-dark schedule. Mice were either wild-type adult (n = 16); wild-type pups, post-natal day 6 or 12, (P6, n = 14; P12 n = 13); or transgenic knockouts that lack the β2 sub-unit of the nicotinic acetylcholine receptor (β2^-/-^, n = 9).

### V1 Tracer Injections

Isoflurane anaesthetised animals underwent a craniotomy exposing V1 (1.5 to 3mm left lateral and -2 to 1.5mm anterior of λ for adult, 1.5–3mm lateral and -1–0mm anterior of λ for P12 neonates, and 1.5–2.5mm lateral and -0.5–0mm anterior of lambda for P6 neonates). Pulled glass pipettes (OD 0.8mm, ID 0.12mm, 11.3nl per mm; tip diameter c. 30 μm), were used to inject fluorescent microspheres (Red, 590nm and Green, 505 nm: Lumafluor, FL, USA; dilution of 1 in 2 with distilled water). After injecting approximately 10–20nl the pipette was withdrawn, the craniotomy closed and the animal recovered. Forty-eight (adults) or twenty-four (P6 and P12) hours later, animals were given an overdose (>0.3 ml) of pentobarbital (Euthatal) and then transcardially perfused with Phosphate Buffered Saline (PBS) and then 4% PFA (paraformaldehyde, in 0.1M phosphate buffer, pH 7.4), and their brains dissected. The following numbers of red or green tracers were used for analysis wild-type adult (Red = 10, Green = 6), β2^-/-^ (Red = 3, Green = 6), P6 (Red = 7, Green = 7), P12 (Red = 5, Green = 8). There was no significant difference in the number of labelled cells from either colour (independent t-test, p = 0.95). In a subset of animals, V1 was sectioned and the volume of injection determined.

### Acquisition of Data and Reconstruction in to a Standardised Space

Brains mounted in 4% agarose were positioned to reproduce the orientation plane of a magnetic resonance imaging (MRI) reference brain (Adult: http://brainatlas.mbi.ufl.edu, neonatal (P6/P12): http://www.birncommunity.org/) and sectioned in 50μm contiguous coronal sections. All sections containing fluorescent label were optically imaged (brightfield and fluorescence; Zeiss Axiophot2 and Axiocam MRm; x10/0.5NA for dLGN and x2.5/0.075NA for V1). Image stitching to reconstruct the whole histological section was done using the FIJI plugin—pair-wise stitching [10]. Images of the dLGN and V1 were manually registered to the MRI reference brain. Distortions associated with sectioning and mounting were corrected with an automated fast-free-form registration [11]. Subsequently, the location of the injection sites in V1 and labelled cells within and boundaries of the dLGN were recorded.

### Analysis

**Fig 1:**
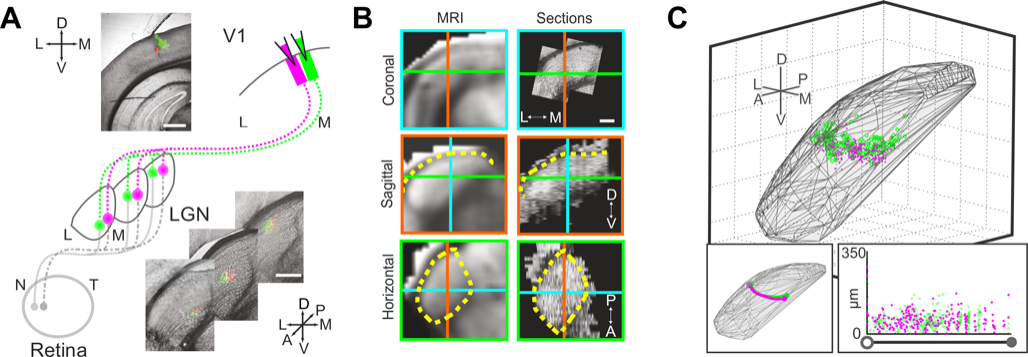
Three dimensional reconstruction of dLGN-to-V1 projection columns. (A) Fluorescent *RetroBeads* were injected into V1 and transported into thalamo-cortical projection neurons within the dLGN. (B) Bright-field images of sections registered to 3D standardized space (MRI). Cross-hairs are colour-coded for each orthogonal view. Yellow dashed lines outline the boundary of the dLGN. (C) Reconstructed locations of labelled somas within the boundaries of the standardized dLGN space (mesh). Left inset: summarised trajectory of the dLGN projection column that extends from the pial surface (⭕) to the ventral boundary of the dLGN (●). Right inset: spread of cells along normalised columns (based on boundary positions: ⭕->●). Scale bars are 250μm.

For each V1 injection, labelled cell coordinates within the standardised dLGN space were used to reconstruct the path of the 3D projection column using a piecewise cubic-spline of order 2 and 5 breaks (Fig 1C). Tracer injections that compromised the white matter tract, missed V1 (lack of labelled cells in the dLGN), or were deemed too large were excluded. A normalised unit column was defined from the intersection of the extrapolated column and the boundary of the dLGN (0 = pial and 1 = ventral end) with 5^th^ percentiles sub-dividing the normalised column (Fig 1C left inset). Analyses of topology and topography: the projection columns within each group were treated as a series of 2D maps, one for each 5^th^ percentile position along each normalised column derived from a best-fit plane to the iso-percentile coordinates along the projection columns. To summarise the trajectory of the projection columns though the dLGN, global expansions and rotations were estimated using a constrained non-linear minimisation algorithm to spatially align neighbouring 2D maps (in-house MATLAB programs). An examination of the internal topological order of the projection columns (i.e. the coherence of neighbouring 2D maps) was determined by calculating the topographic product (P_t_)–a graph based metric for quantifying the relative order between multiple pairs of points [12]. Topographic product provides an estimate of order across all scales of the map by examining the ratio of distances in each space (neighbouring 2D maps) for all pairs of points. Here we have modified the topographic product by normalising it to the P_t_ of the permuted map. In this way, the normalised P_t_ ranges between 1 (complete disorder) and 0 (perfect order). In a similar way, the topographic order between the V1 coordinates of injection sites and the corresponding columns within the dLGN were determined between V1 and each matched 2D dLGN map along the length of the column. To estimate the number of latent variables (independent contributions to the metrics describing 3D projection columns), a partial least squares regression approach with a 9-fold partition cross-validation was used. All analyses were custom written in MATLAB.

## Results

### Labelled and reconstructed dLGN projection columns

Focal injections of retrograde tracer into V1 revealed patches of labelled cells within the dLGN over several serial histological sections (Fig 1A). Ex-vivo MRI provided 3D dLGN reference spaces to guide manual alignment of histological sections in order to faithfully follow the curvature of the dLGN (Fig 1B). Together with subsequent automatic image processing to correct for local deformations, these produced high quality 3D reconstructions of dLGN projection columns (Fig 1C). Once reconstructed, the path of each projection column was summarised to reveal its trajectory (Fig 1C–left inset). Distributions of cells relative to the splined path of the projection columns could then be derived and normalised relative to the pial and ventral ends of the columns (Fig 1C–right inset).

### Pooling and comparing projection columns

**Fig 2:**
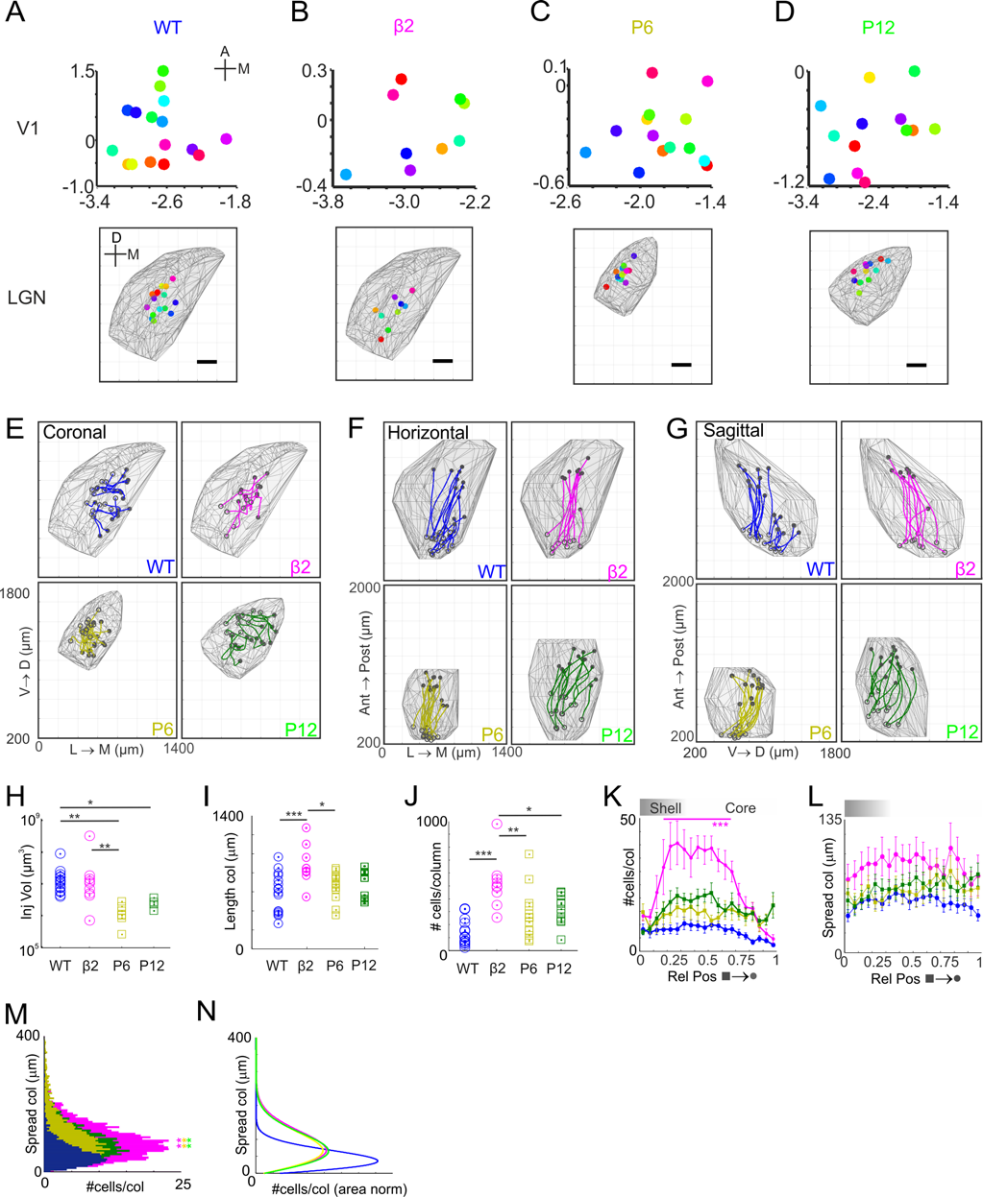
Form and Features of dLGN-V1 Projection Columns. (A-D) Location of V1 injections (mm relative to Lambda) and positions of the labelled column on the pial surface (scale bar 200μm) for four groups: Adult Wild Type (WT), n = 16; Adult β2^-/-^, n = 9; P6 WT, n = 14, P12 WT, n = 13. Coordinates are relative to lambda. (E-G) Reconstructed paths of dLGN-V1 projection columns collapsed onto coronal (E), horizontal (F), and sagittal (G). Columns extend from the pial surface (⭕) to the ventral boundary of the dLGN (●). (H) Distribution of injection volumes in each group, Kruskal Wallis test, with Dunn’s Multiple Comparison Post Hoc Tests, *p<0.05, ** p<0.01. (I) Column lengths. (J) Number of cells per column. (I & J) One-way ANOVA with Bonferroni post-hoc tests: * p<0.05; ** p<0.01; *** p<0.001. (K) Number of cells per 5^th^ percentile of unit column. Data presented as mean ±SEM. Grey shading in this and subsequent figures indicate the shell core boundary at approximately 30% of the projection column. Two-way ANOVA with Bonferroni post-hoc tests relative to pial end: *** p<0.001. (L) Spread of cells from the centre of mass of each column per 5^th^ percentile. Data presented as mean ±SEM. Values are corrected for different sized dLGNs by scaling to the WT dLGN. (M) Cumulative histogram illustrating the numbers of cells of a given spread (see L). Kruskal Wallis test, with Dunn’s Multiple Comparison Post-hoc tests compared to WT: ** p<0.01. (N) Fitted Rayleigh distributions to cumulative data in M, normalised to the area under each curve.

Registering labelled cells from multiple histological sections within group-appropriate dLGN standardized spaces enabled data from multiple projection columns and animals to be pooled. Projection columns in four separate groups across normal or perturbed development were examined: adult wild-type; neonatal wild-type (P6 and P12); and adult β2^-/-^ mice. All V1 Injections within each group are summarised in Fig 2A–2D with respect to the point at which the labelled dLGN column intersects with the pial surface. Fig 2E–2G illustrates the complex yet relatively coherent summary trajectories of projection columns through each group’s dLGN standardized space. The WT adult group exhibited a number of shorter columns within the posterior-ventral pole (best seen in Fig 2G). This reflects the greater scatter of injection sites across V1 in the WT group (Fig 2A–2D). Injection volumes (where calculated) are provided in Fig 2H. While the injections are of a smaller volume in both P6 and P12 when compared to the WT (Kruskal-Wallis with pairwise comparisons, p<0.03), there is no significant correlation between the volume injected and the number of cells labelled (Spearman’s Correlation, p = 0.732).

Given the range of column lengths within and across groups and numbers of cells per column (Fig 2I and 2J), a unit column from the pial surface (0 –shell) to the ventral border (1 –deep core) was devised to enable the pooling of information across columns. Collating the number of cells within each 5^th^ percentile of length along the unit column revealed a number of features. Firstly, there is a main effect of group wherein all groups exhibit a statistically significant different number of cells within a column (Fig 2J). Secondly, the distribution of cells within the WT adult group is near-uniform with a gradual drop in cell numbers towards the deepest core (Fig 2K). Thirdly, the P6, P12 and β2^-/-^ groups exhibit a more complex distribution of cells within the middle portion of the unit projection column with a clear transition between shell and core regions particularly for the P12 and β2^-/-^ groups. The β2^-/-^ group also exhibited a statistically significant larger number of cells in the middle half of the column (15% to 65% of the column, p<0.001, 2-way ANOVA with Bonferroni post-hoc tests). Fourthly, there is a subtle difference between the P6 and P12, and adult groups over the deepest section of the core (0.9–1).

By calculating the perpendicular distance of each labelled cell from column trajectory, we can assess the spread of cells–note corrections for dLGN volume differences between groups were applied. While there is a main effect of group (Fig 2L, F = 28.24, p<0.01, 1 way ANOVA with Bonferroni Post-Hoc tests) with the WT adult group exhibiting less spread of cells compared to all other groups, there was no interaction with column location (2-way ANOVA, p = 0.87). Collapsing the column (Fig 2M) confirms the increased spread in the developmental and β2^-/-^ groups relating to WT. While the overall spread of cells within the β2^-/-^ group appears greater than all groups, it is a consequence of the larger number of labelled cells (Fig 2J) and the inherently asymmetric Rayleigh distributions associated with cell spread (Fig 2M). Correcting for the number of cells within group (Fig 2N) confirms the equivalent spread of cells in the P6, P12 and β2^-/-^ groups.

### Topological and topographical order

**Fig 3:**
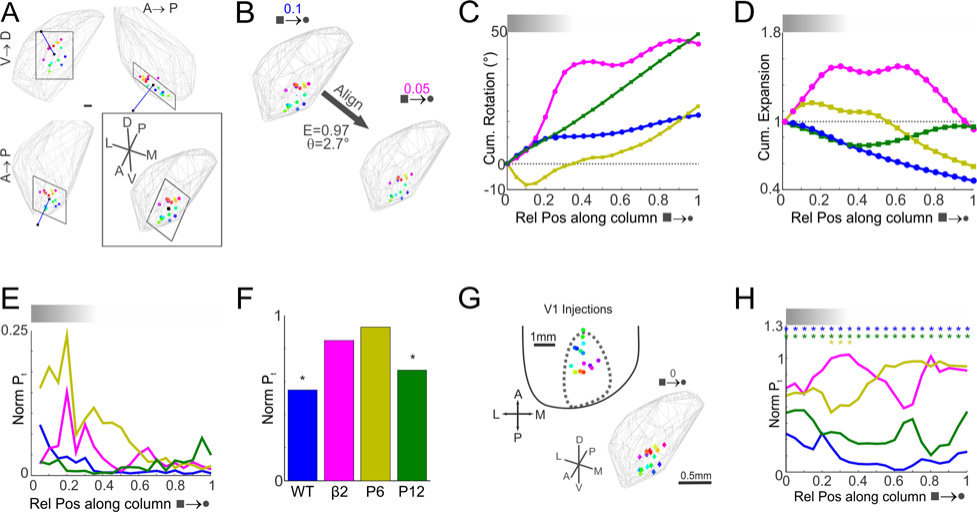
Dynamic Topological and Topographical Order in Geniculo-Cortical Projection Columns. Generating maps of dLGN columns for each 5^th^ percentile along its length enables estimations of organisation and order. (A) Example of a best-fit plane (normal vector: blue-line) for WT projection columns at 10^th^ percentile (0.1) position. Inset: collapsed view orthogonal to the plane–end on normal vector. Scale bar is 100μm. (B) Example of registration (expansion—E; rotation - θ) between neighbouring (0.1 and 0.05) WT planes. (C) Cumulative rotation and (D) expansion of each 5^th^ percentile map aligned to the pial plane (0). (E) Internal topological order within each group compared to the preceding 5^th^ percentile position. Note, a normalised P_t_ of 1 represents complete disorder while 0 is perfect order. (F) Degree of topological order between the pial and ventral maps of the dLGN. Monte Carlo permutation tests: * (p<0.05)—statistically significant order. (G) Schematic: correspondence of WT V1 injection sites to dLGN pial plane (0). (H) Degree of topographical order between V1 and the dLGN at each 5^th^ percentile. Monte Carlo permutation tests: * (p<0.05)—statistically significant order.

The reconstruction of columns within standardized space enabled an assessment of the paths of the projection columns through the dLGN (Fig 3A–3D); their relative internal topological order (Fig 3E and 3F) and topographic order in relation to V1 (Fig 3G and 3H). The path of the projection columns and internal topological order were assessed by comparing neighbouring 5^th^ percentile locations along the unit column. By deriving a best-fit plane, a 2D map representing the relative arrangement of columns at each 5^th^ percentile position along the unit column could be determined (Fig 3A). Neighbouring maps were spatially aligned (Fig 3B) to estimate the global expansion and global rotation of the projection columns as they traverse the dLGN. A diverse range of rotations and expansions of the columns are revealed within and across different ages (Fig 3C and 3D respectively). In particular, the rotations of the columns within the early age group (P6) appear to exhibit marked differences compared to all other groups suggesting a marked developmental transition between P6 and P12. Indeed, at shallower (close to pial surface of dLGN) regions (~0–0.4 of the unit column) the WT and P12 groups are almost identical. Additional, the trajectory of the columns for the adult β2^-/-^ columns appear to be markedly different from those of the WT adult group. Whilst this approach yields summary information about the differential trajectories of the dLGN column, it does not evaluate the preservation of order.

The topological product was determined for matched columns across neighbouring 2D maps along the length of the projection columns. The calculated normalised topographic product represents the degree of preserved order, with a value of zero representing perfect order and one representing complete disorder. Once again, the degree of topological order between neighbour locations along the length of the dLGN columns exhibits a diverse range of internal topology (Fig 3E). The P6 and adult β2^-/-^ groups appear to exhibit greater disorder in the more superficial third of the dLGN, whilst there is more coherent organisation in the deeper regions of the dLGN. By comparing the topological order between the pial and ventral planes (0 and 1 respectively) of the dLGN projection columns the degree of preserved topological order between the two ends of the columns could be compared (Fig 3F). Both the WT and P12 exhibit statistically significant preserved order between the pial and ventral limits of the projection columns (p<0.001 and p = 0.0025 respectively), whilst the P6 and adult β2^-/-^ groups both failed to exhibit greater order than chance (p = 0.28 and p = 0.19 respectively). Note p-values represent the probability of finding an equally ordered map by randomly permuting one map many times (Monte-Carlo simulation)

The topographic organisation of the labelled dLGN columns with respect to the V1 injection sites (Fig 3G) was similarly assessed at every 5^th^ percentile position along the dLGN column (Fig 3H). Topological product measurements again demonstrate a diverse degree of topological order within and across groups with respect to V1 (Fig 3H). The WT and P12 groups are in closest agreement, exhibiting statistically significant order with respect to V1 across the entire column length (p<0.05). In comparison, the adult β2^-/-^ group exhibits no topographic order with V1. Whilst the P6 group is generally topographically disordered at this age bar a small zone between 0.25 and 0.35 that just reaches statistically significant order (p<0.05) with respect to V1.

### Explaining columnar variance–the number of latent variables

**Fig 4:**
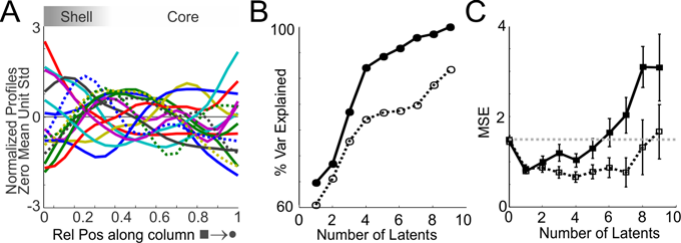
PLS Regression Suggests Complex Cryptic Organisation. (A) Normalised profiles (zero mean and unit variance) representing the numbers of cells; spread of cells and normalised P_t_ representing topological and topographical order for all groups (WT, P6, P12 and β2^-/-^). Colour coding is arbitrary except that the β2^-/-^ group are dashed lines. (B) PLS regression; the degree of variance within A is increasingly explained by an increasing number of latents. Curves represent the explained variance for the combined WT, P6 and P12 groups (solid line) and all groups combined together (dashed). (C) Cross-validation error (Mean Squared Error—MSE) as a function of the number of latents retained in the model. WT, P6 and P12 groups (solid line) and all groups (dashed).

The reconstruction of columns of dLGN projection neurons to V1 within a standardized space has enabled data to be pooled, enabling assessments of parameters that define various features of the projection columns. This approach has revealed a surprising degree of diversity in a number of measures (Figs 2J, 2K, 3C and 3D and 3F) across all groups. It is intriguing to speculate whether such diversity reflects differential and independent developmental factors along the column. Further, do such independent factors represent independent but overlapping laminar architecture—cryptic lamination? If so, how many putative laminae would account for the variance observed? Using a partial least squares (PLS) regression approach, we determined the number of independent factors (latents) that account for the variance in the data (Fig 4A). Note, as each metric has its own scale and relative gain, only the shape of each parameter was used by applying a shape preserving normalisation–zero mean, unit variance. Using the PLS approach determined the number of variants that explain the variance across normalised parameters (Fig 4B). Due to the unknown effect of altered retinal drive (β2^-/-^ group) on ‘normal’ dLGN organisation, two PLS analyses were determined: one with only wild type groups (blue line, Fig 4B) and another including all groups (magenta line, Fig 4B). In either analysis the percentage of explained variance was considerably higher for >2 latents, suggesting the shell/core may only account for ~65% of the variance. Although, increasing the number of latents will account for greater amounts of variance it is usually specific to the dataset and incrementally represents over-fitting. By incorporating a cross-validation test using a leave-one-out strategy the mean standard error associated with repeated omissions provides an estimate as to the confidence regarding the actual number of latents (Fig 4C). Taken together, the explained variance and prediction error (Fig 4B and 4C) suggests 4–6 latent variables may account for the diversity of projection columns observed in the wild type groups. Further, it may predict that the β2^-/-^ group could disrupt the number of independent latents.

## Discussion

By employing 3D reconstruction techniques and placing datasets from multiple subjects, across different ages and genetic backgrounds into common standardized spaces, we have been able to study the developing thalamo-cortical projection. Pooling data in this way has revealed the meso-scale architecture of dLGN-V1 projection columns, and how collectively the internal arrangement (topology) and organisation with reference to V1 (topography) change along the length of the column.

Collectively, our observations revealed a surprising diversity within and across columns in terms of the number and spread of cells within a column; the trajectory and topology between columns; and topography relative to V1. In particular, large variances have been demonstrated along the length of projection columns in young animals. Such variance appears reduced in the adult group, with residual differences being consistent with the superficial shell-core zones [6]. Could the variances observed within and across the projection columns of young animals transiently reflect the cryptic lamination of the dLGN through different developmental dynamics between lamina? While additional laminae have not been demonstrated here or elsewhere; analysis of the number of latent variables that may account for the observed variation across the length of the column suggest that there may be approximately 4–6 factors. Whether these represent 4–6 cryptic lamina is beyond the scope of this study to confirm but, if correct, it suggests a surprising degree of compartmentalisation of visual information within the mouse dLGN. Furthermore, including data from the β2^-/-^ knock-out group in the PLS regression suggests a disruption of stage II cholinergic waves may substantially disrupt the cryptic organisation of the dLGN, indicating that the β2^-/-^ mutation disrupts far more than previously reported [13]. It should be noted that, while unlikely, the diversity observed within and across columns may be a result of experimental variability in the size of injection volumes and distribution of V1 injection sites.

In other animals such as cats the dLGN has a clear role in modulating the strength of geniculo-cortical outputs, and receives inputs from other cortical regions [14]. However, historically the mouse dLGN was thought to be an amorphous nucleus that behaved as a simple one-2-one relay station between the eye and cortex. However, with the expansion of mouse studies over the past decade suggestions of cryptic lamina are growing. In particular, the shell-core arrangement appears prominent [3,6] and, reports of different RGC sub-types innervating different portions of the dLGN suggest the possibility of even more laminae [1,2,15]. These cryptic lamina are not restricted to the retino-geniculate projection, geniculate projection neurons also appear to have a distinct morphology that is dependent on their location within the dLGN [16]. Furthermore functional differences between lamina have been demonstrated in the rat [17], and regions (though not strictly lamina) have been shown in the mouse itself [5]. It is not beyond the realms of possibility for there to be multiple missed laminae. Such emergent laminae have been discovered in areas that were previously thought to be simple, e.g. the zebrafish optic tectum [18] and the SC [19,20].

Recent advances in optical clearing techniques [21–23] are facilitating 3D assessments of large and deep tissues however conformational changes to the tissue compromises anatomical fidelity. Further, the lack of reference anatomy limits the utility of such techniques. There are several advantages to reconstructing histological slices into a 3D reference space; but principally it enables pooling of data derived from multiple subjects. Therefore, techniques that only sample a small object in single animals can be used to derive population information across many subjects, revealing previously inaccessible anatomical and functional organisation. It should be noted that further research and development is required to fully automate this process to enable efficient data collection. Particularly, automated section-image cryostat-confocal imaging would significantly improve though-plane resolution and image registration fidelity.

How and why parallel streams of sensory information are organised as unsegregated, cryptic or overt lamination is not clear. It is particularly unclear as to whether overt and cryptic lamination represent fundamental differences in computation. The distinct lamination in carnivore and primate dLGN ensures co-compartmentalisation of retinal inputs and projection neurons. Yet, does the suggested cryptic lamination of projection neurons, extending beyond shell vs core serve a similar function in mouse dLGN? In which case, one prediction would be a co-compartmentalisation not only of retinal inputs but also of the dendritic arbours of projection neurons. A study of dendritic morphology mouse dLGN neurons [16] indicates that this is true for W-like neurons, which are confined to the geniculate shell. It would be interesting to examine the orientation of the X-like neuron arbours with respect to the local orientation of the projection column.

Our reconstructed paths of columns through the dLGN could also provide a guide for future electrophysiological studies. Further, collating the findings of many independent functional studies within a standardised dLGN space will enable a cumulative map to be derived. Population maps of localised functional parameters will have the power to reveal or refute subtle overlapping differential components or emergent computation of visual scene processing along the dLGN-V1 projection column. To this end we have made our standardized spaces of projection column pathways, analysis protocols and matlab scripts publically available (doi = 10.6084/m9.figshare.2008698).

Our reconstruction and analysis techniques highlight the possibility of significantly greater underlying organisation within the mouse dLGN as revealed by complex developmental variances in the thalamo-cortical projection columns. Further, we demonstrated a potential role for patterned activity. These are surprising findings that suggest the organisation of visual information within the mouse dLGN may be more complicated than previously thought and warrants significant future research to probe and dissect the functional properties along the column. We believe our findings and approach will be of significant utility in this regard.
